# Endophytic *Fusarium oxysporum* GW controlling weed and an effective biostimulant for wheat growth

**DOI:** 10.3389/fpls.2022.922343

**Published:** 2022-08-05

**Authors:** Syed Asim, Anwar Hussain, Waheed Murad, Muhammad Hamayun, Amjad Iqbal, Hazir Rehman, Abdul Tawab, Muhammad Irshad, Abed Alataway, Ahmed Z. Dewidar, Hosam O. Elansary, In-Jung Lee

**Affiliations:** ^1^Department of Botany, Abdul Wali Khan University Mardan, Khyber Pakhtunkhwa, Pakistan; ^2^Department of Food Science and Technology, Abdul Wali Khan University Mardan, Khyber Pakhtunkhwa, Pakistan; ^3^Department of Microbiology, Abdul Wali Khan University Mardan, Khyber Pakhtunkhwa, Pakistan; ^4^National Institute for Biotechnology and Genetic Engineering, Faisalabad, Pakistan; ^5^Prince Sultan Bin Abdulaziz International Prize for Water Chair, Prince Sultan Institute for Environmental, Water and Desert Research, King Saud University, Riyadh, Saudi Arabia; ^6^Department of Agricultural Engineering, College of Food and Agriculture Sciences, King Saud University, Riyadh, Saudi Arabia; ^7^Plant Production Department, College of Food & Agriculture Sciences, King Saud University, Riyadh, Saudi Arabia; ^8^Department of Applied Biosciences, Kyungpook National University, Daegu, South Korea

**Keywords:** bioherbicide, biofertilizer, phytohormones, antioxidants, *Fusarium oxysporum*, endophytic fungi

## Abstract

Wheat crop has to compete with several weeds including *Avena fatua*, a noxious weed that alone is responsible for 30–70% losses in the yield annually. Because of the environmental concerns associated with conventional methods, researchers are on a continuous hunt to find clean alternatives in order to manage weeds. Fungi have shown promising weedicide potential in lab studies. The current study aimed to isolate endophytic fungi from wheat plants which can promote wheat growth and inhibit the growth of common weed, *A. fatua*. Of several isolates, GW (grayish white) was selected for its promising features, and the strain was identified as *Fusarium oxisporum* through ITS sequencing technique. This fungus released a number of compounds including Isovitexin, Calycosin, quercetagetin, and dihydroxy-dimethoxyisoflavone that inhibited the growth of *A. fatua* but did not influence the growth of wheat seedlings. Biomass of this fungus in the soil also reduced growth parameters of the weed and promoted the growth of wheat. For instance, the vigor index of *A. fatua* seedlings was reduced to only 6% of the control by this endophyte. In contrast, endophyte-associated wheat seedlings showed a higher vigor index than the control. Behind this differential response of the two plants were their contrasting physiological and biochemical status. Lower growth phenotypes of *A. fatua* seedlings had reduced levels of IAA, GAs, and SA and higher the levels of JA and ABA. Besides, their ROS scavenging ability was also compromised as evident from relatively lower activities of catalase, peroxidase, and ascorbic acid oxidase, as well as higher accumulation of ROS in their leaves. Wheat seedlings response to GW was opposite to the *A. fatua*. It may be concluded that *F. oxysporum* GW has the ability to differentially modulate physiology and biochemistry of the two hosts leading to contrasting phenotypic responses.

## Introduction

*Triticum aestivum* L. (Wheat) is one of the important cash crops grown as a source of food worldwide. According to Government of Pakistan (GOP, 2013), agriculture contributes 2.2% to the total Gross domestic product (GDP) of the country out of which wheat makes 10.1%. In wheat yield, weed-induced reduction is a major contributing factor responsible for below-average yield of wheat (Hussain et al., 2007). Weeds are among the undesired plants growing in the field alone with crops, competing for nutrients and space and affecting the yield of the crops (Javaid et al., 2007). Also, weed may host devastating pathogens responsible for the attack on crop plants and reducing their yield.

*Avena fatua* (wild oat) is among the top 10 most devastating weeds in the temperate regions, and this weed alone is responsible for 17–70% yield losses in winter wheat (Beckie et al., 2012; Dahiya et al., 2019). The decline in wheat yield is directly proportional to the density of the wild oat. Wheat yield loss is <1% in field having up to 3 wild oats/m^2^, reaching 2.2% in 5 plants and 50–60% in 100 wild oats/m^2^. A number of synthetic herbicides including clodinafop-propargyl, fenoxaprop, accord plus (binary combination of fenoxaprop and metribuzin), isoproturon, atlantis (binary combination of meso and iodosulfuron), pinoxaden, and sulfosulfuron are available to protect crop plant for this weed. However, herbicide resistance in wild oat is a growing concern. Excessive use of synthetic herbicides is another important concern regarding the safety of sustainability of the environment. To address these issues, the use of microorganism, obtained from the environment, offers an ecofriendly alternative for sustainable weed management (Saxena and Pandey, 2001; Batish et al., 2002; Beckie et al., 2012).

Several fungi have been shown to possess bioherbicidal potential and can be used for weed management (Bailey, 2014). However, most of the fungi used as bio-herbicide are pathogens because of their high specificity and non-dependence over the vector for dissemination. For this purpose, obligate biotrophic pathogens are mostly preferred because of their ability to inflict severe damage on a host by feeding directly on living tissues. The target plant is rarely killed but imposes nutrient drain and wastage of energy which is harmful to plants (Charudattan, 2005). It is important to know that once deployed in the environment, the microorganisms can't be controlled. In this connection, endophytic fungi may be more suitable, which can inhibit weed growth and simultaneously promote the growth of the crop plant, because of their dual benefits. Endophytic fungi often go unnoticed because of their asymptomatic presence in the host plant (Wilson, 1993). However, there is compelling evidence suggesting a strong effect of endophytes on the composition and productivity of plants in natural and agro-environments. Previous studies have shown that endophytic fungi can be effective in controlling weeds (Saikkonen et al., 2013).

Endophytic fungi may control weed in a number of ways, affecting seed germination and later stages of growth and development. For instance, fungal metabolites may be absorbed by weed seeds resulting in damage to the cell membrane, activity of amylase, cell division, and other vital processes, resulting in delayed or inhibited seed germination. Also, they may grow in the intercellular spares of roots, releasing toxic metabolites, and inhibiting seed germination. Another reason for compromised seed germination and weed growth is suppressed photosynthesis and phytohormones activities, resulting in enhanced accumulation of ROS and stress hormones including ethylene and abscisic acid (Radhakrishnan et al., 2018).

Besides their bioherbicidal potential, endophytic fungi are known to improve the growth of the host plant in a number of ways. In this connection, the production of phytohormones is the most studied aspect of endophytic fungi (Hamayun et al., 2010). A high level of IAA and low level of ethylene increase root and shoot length, as well as weight of the plant (Glick et al., 2007). Endophytic fungi also have the ability to mobilize nutrients such as phosphate and calcium, enhancing their availability to the host plant (Spagnoletti et al., 2017). The release of Siderophores by the endophytic fungi not only contributes to host growth by improving iron availability but also keeps pathogenic fungi away (Ripa et al., 2019). Other useful properties of endophytes on plants consist of nitrogen fixation, increased drought resistance, thermal protection, and persistence under osmotic stress (Khan and Doty, 2009). Based on the benefits of endophyte association with the crop plants, the use of endophytic fungi as biofertilizers is extensively researched (Ripa et al., 2019).

The current study aimed to isolate endophytic fungus from vigorously wheat genotypes that can inhibit the growth of the noxious weed (*A. fatua*) and simultaneously promote the growth of wheat plant. To understand the contrasting effect of the fungus on wheat and weed, physiological and biochemical responses of the plants were studied.

## Methodology

### Endophytic fungi isolation

Healthy and vigorously growing plants were collected from different cultivated fields of Mardan (Pakistan) and processed in the PMI laboratory, located in Botany department, Abdul Wali Khan University Mardan. The collected plants were cleaned by washing and then their leaves, stem, and roots were separated. The parts were cut into segments (2 cm long), which were then sterilization of surface with 70% ethanol for 30 s. Autoclaved distilled water was used to remove the ethanol from plant materials (Arnold et al., 2001). The sterilization of surface segments were injured with sterilization of scalpel to expose internal tissues. Hagem agar (containing 8 μl streptomycin) was used to incubate the explants. Control plates received imprints of the sterilization of surface of explants. The plates were maintained in an incubator set at 30°C. The colonies sprouting out the explants tissues were transferred to potato dextrose agar (PDA) plates and purified by repeated sun cultures. The axenic cultures were maintained at 4°C for further use.

### Screening the isolates for weedicide potential

The isolates were grown in Czapek broth at 130 rpm for 5 days under the culture conditions mentioned above (Mehmood et al., 2018). Fungal biomass and culture filtrate were separated by filtering the culture by the end of incubation time.

Surface of the healthy and uniform seeds of wheat cultivar Faisalabad-2008 and *A. fatua* was sterilized by keeping the seed in 0.1% HgCl_2_ for 1 min. The seeds were then rinsed with autoclaved distilled water (thrice) and placed on double-layered wet Whatsman No. 1 filter paper. The treatment plates received culture filtrate of endophytic fungi isolated from wheat and control plates received blank Czapek medium. The plates were at room temperature receiving artificial light for 8 h a day. The growth of the seedlings was monitored for 7 days, and the impact on growth of the seedlings was recorded as growth promotion or inhibition by comparing seedling's length with the control seedlings. Each treatment was applied to five plates and each plate contained 5 seedlings. The experiment was repeated two times.

### Selection of fungal endophytes

The isolates, capable to promote the growth of wheat seedlings and inhibit the growth of *A. fatua*, were selected for further study.

### Identification

For identification purposes, the selected endophytes were grown in slide culture (Tiwari, 2012), and the cultures were then observed under light microscope to study the morphology of the isolates. To further confirm the identity of the isolate, fungal biomass was processed for genomic DNA isolation using SolGent Fungal DNA Extraction Kit (Cat No. SGD64-S120; SolGent Co., Daejeon, Korea) (ITS1) as described by Waqas et al. (2012). The genomic DNA was used as template to amplify internally transcribed spacer sequence (ITS) of 18 S rDNA using NS1 5′ (GTA GTC ATA TGC TTG TCT C) 3′ and NS2 5′ (AAA CCT TGT TAC GAC TTT TA) 3′ (ITS4 universal) primers. The PCR amplified product was sent to DNA sequencing service of MACROGEN, Korea (http://dna.macrogen.com/eng) for sequencing. A consensus sequence was made of the two reads by aligning the reads in Codon Code Aligner (version 7.2.1, Codon Technology Corporations). The sequence obtained was then processed for identifying similar sequences through nucleotide BLAST using NCBI GenBank database (https://blast.ncbi.nlm.nih.gov/Blast.cgi?PAGE_TYPE=BlastSearch). Closely resembling sequences in the database were retrieved and used to make a phylogenetic tree through MEGA11 software (version 11.0.11). The neighbor-joining tree was made by setting all the options at default except test of phylogeny and gap/missing data treatment which were set to partial deletion and bootstrap method, respectively. The ITS sequence of *Atkinsonella hypoxylon* (U78051) was used as an out-group.

### Characterization of the selected endophyte

The selected strain was grown in 50 ml Czapek broth contained in a 500-ml round bottom flask. The flasks were maintained at 28°C and 120 rpm for 5 days. After the completion of incubation period, fungal biomass was separated from broth by filtration using Whatsman No. 1 filter paper. The experiment was performed under sterilized conditions in a laminar flow hood using autoclaved glassware and filter paper. The biomass was saved in flacon tubes at 4°C for future use. Culture filtrate of the selected isolate was characterized by the presence of different metabolites.

#### Phytohormones profiling

Profiling of phytohormones including indole-3-acetic acid (IAA), gibberellins (GAs), salicylic acid (SA), abscisic acid (ABA), and Jasmonic acid (JA) was done through HPLC (PerkinElmer, USA) equipped with UV/Vis detector, isocratic pump, and a reverse phase C18 column (250 × 4.5 mm). The culture supernatant obtained fungal culture grown for 5 days in Czapek medium was filtered through mentioned phytohormones. To extract phytohormones, fungal culture filtrate was shaken with equal volumes of methanol:acetic acid (80: 1, v/v/v) in the dark at 4°C for 16 h in dark. The organic phase was taken as phytohormones extract. The extracts were separately passed through syringe filtration using Nylon 66 filter (13-mm diameter and pore size 0.22 μm; Jinteng Experiment Equipment Co., Ltd, Tianjing, China) to exclude suspended particles. The extract (20 μl) was injected onto a reverse phase C18 column using 80% methanol at a flow rate of 1 ml/min for IAA.

Gibberellin (GA_3_) was eluted with 20% acetonitrile taken in ammonium dihydrogen phosphate (5 mmol l^−1^) at a flow rate of 0.6 ml min^−1^. pH of the solvent was adjusted to 2.5 with H_3_PO_4_. The eluate was monitored at 205 nm to identify GA_3_. Standard GA3 (Sigma) was used to make a calibration curve for comparison (Lale et al., 2006).

Elution of ABA was carried out at 1.0 ml/min, using a solvent composed of 0.6% methanol and acetic acid (mixed in 6:4 v/v; Gupta et al., 2011).

Elution of SA was performed by passing the mobile phase prepared by mixing acetonitrile, methanol, and NaH_2_PO_4_·H_2_O (pH adjusted to 5.5 with 1N NaOH) in 65:6:29 v/v ratio (Ref).

Elution of JA was performed with a mixture of methanol and 0.2% aqueous acetic acid (1:1 v/v). The flow rate was maintained at 1 ml/min.

The eluates were separately passed through a UV detector set at 280, 205, 275, 254, and 195 nm to scan for IAA, GAs, SA, ABA, and JA, respectively. For quantification, different concentrations (0.1, 1, 10, 100, and 1,000 μg/ml) of the reference compounds were analyzed under the abovementioned conditions to obtain a standard curve.

#### Determination of phenols and flavonoids

Total phenols and flavonoids were determined in fungal culture filtrate by using colorimetric method described previously (Chaiyana et al., 2020). For the determination of flavonoids, fungal culture filtrate (500 μl) was mixed with a 10% solution of aluminum chloride (100 μl), 10% potassium acetate (100 μl), and 80% methanol (4.8 ml). The reaction mixture was vortexed and incubated for 30 min at room temperature. The colored product was quantified by taking Optical density (OD) at 415 nm. A standard curve was prepared by using standard quercetin (10 μg−100 μg/ml).

The fungal culture filtrate was subjected to total phenols determination through the Folin–Ciocalteu method with slight modification. To prepare reaction mixture, 0.2 ml of fungal culture filtrate, 2 ml of 7.5% Na_2_CO_3_, and 0.8 ml of Folin–Ciocalteu reagent (FCR) were taken in a test tube. Distilled water was used to dilute the reaction mixture 7 times, and the diluted mixture was incubated for 2 h in dark at room temperature. For comparison and quantification, standard catechol was used. The resulting color compound was quantified by monitoring its absorbance at 765 nm against a blank. Catechol concentration (1 mg−10 mg/ml) was used for making reference standard curve.

#### Fractionation of fungal culture filtrate

Fungal culture filtrate was fractionated by eluting a 100 μl sample from a reverse phase column through 70% methanol as a mobile phase. The eluate was monitored through UV detector as described above. Different peaks were collected through a fraction collector. Fractions were pooled from five rounds of fractionation, and the pooled fractions were concentrated on a rotary evaporator under vacuum. The fractions were tested for weedicide potential by testing the water reconstituted fraction on *A. fatua*. The active fractions were subjected to Liquid chromatography equiped with mass spectrometry (LC-MS/MS) for identification of the active components.

#### Mass spectrometry of HPLC fractions

The active fractions were directly injected to ESI-MS/MS (LTQ XL, ThermoElectron Corporation, USA) as described previously (Mehmood et al., 2019). The injected samples were fractionated through methanol and acetonitrile [80:20 (v/v)]. The mass ranges, in positive and negative ionization (wherever required) mode, were chosen from 50 to 1,000 m/z. During MS/MS, the collision-induced dissociation energy (CID) varied from 10 to 45, according to the parent molecular ions produced. The solvent flow rate was adjusted to 8 μl/min at a capillary temperature 280°C. The MS parameters were adjusted to achieve maximum favorable ionization, ion transfer conditions, and optimal signal of both the precursor and fragment ions. The data obtained were evaluated using a manual, Xcalibur (Xcalibur 3.0). The structure elucidation was performed in ChemDraw (ChemDraw Ultra 8.0), and the mass scans were compared with online data to identify the compounds present.

### Plant interaction experiment

Uniform healthy seeds of wheat and *A. fatua* were sown in plastic pots containing 100 g autoclaved garden soil. To the soil, fungal biomass was added at the rate of 1 g of fresh biomass per 100 g soil. Two different experiments were performed using a completely randomized design. The first experiment used wheat seedlings, and the second experiment used *A. fatua* seeds. In each experiment, the pots were divided into two sets in order to receive two levels of the treatment (endophytic fungus GW and control). There were five pots per treatment and the experiment was repeated three times. The pots were incubated in a plant growth chamber (DAIHN Lab Tech) under controlled conditions (photoperiod 11/13 hat at 18°C at day and 15°C at night; 390 ppm CO_2_; 40% humidity) for 21 days. Germination (%), mean daily germination, and seedlings vigor index were recorded. The seedlings were harvested and immediately processed for measuring different parameters or frozen in liquid nitrogen and stored at −80°C.

#### Growth parameters of the seedlings

Root length and shoot length were recorded by using a measuring tape. The emergence of embryonic axis (radicle) from the seeds was scored as seed germination. Seedlings vigor index (VI) was determined by using the formula, VI = seedling's length × germination (%). Leaf relative water content (RWC) was measured according to González and González-Vilar (2001) using the following equation:

Leaf RWC (%) = [(FM-DM)/(TM-DM)] × 100. Electrolyte leakage of the leaves was determined after removing the surface adhered electrolytes by washing the leaves three times with deionized water. Disks of equal size were scraped out of the leaves were then incubated in 10 ml dH2O at 120 rpm and 25°C for 24 h. Distilled water containing leaked electrolytes was analyzed for electrical conductivity (L1), and the disks were autoclaved for 20 min at 120°C to estimate electrical conductivity (L2). The percent leakage of the electrolytes was measured by using the following formula (Irshad et al., 2021):


(1) $$\begin{array}{r} \text{EL}=\text{L1/L2}\times 100 \end{array}$$

#### Determination of antioxidant potential of the seedlings

The following activities were performed to measure antioxidants in the leaves.

#### DPPH (2,2-diphenyl-1-picrylhydrazyl) scavenging activity

To determine the antioxidant potential of the leaves, DPPH-scavenging activity was performed (Hussain et al., 2020). Sample was prepared by grounding 0.1 g of frozen leaves, and the fine powder was added to 1 ml of methanol. The DPPH solution was made in methanol and its final concentration was adjusted to 0.004%. The sample was filtered and mixed with DPPH solution at 1:2 (v/v). The mixture was left in the dark for 30 min at room temperature. Color fading was observed by taking OD of the reaction mixture at 517 nm. Percent inhibition of DPPH was calculated by using the previously developed formula:


(2) $$\begin{array}{r} \%DPPH=\frac{\left(1-AE\right)}{AD}\times 100 \end{array}$$

AE = OD of the reaction mixture (Sample + DPPH)

AD = OD of DPPH solution without sample added.

#### Catalase activity determination

The crude enzyme extract was prepared by adding 0.5 g of frozen leaves sample to 25 mM potassium phosphate buffer (pH 7.8) composed of 1 mM ascorbate, 0.4 mM EDTA, and 2% (w/v) polyvinylpolypyrrolidone. The mixture was shaken at 15,000 rpm for 1 min to homogenized its contents, and debris were removed by centrifugation for 20 min at 15,000 g. The supernatant was passed through syringe filtration to clear the crude enzyme extract. To the crude enzyme extract, 50 mM potassium phosphate buffer and 10 mM H_2_O_2_ were added. The activity of catalase enzyme was assayed by monitoring H_2_O_2_ decomposition rate at 240 nm (Aebi, 1984).

#### Peroxidase

Dehydrogenation of guaiacol was monitored for the quantification of peroxidase activity (Malik and Singh, 1980; Bukhari et al., 2021). To prepare crud enzyme extract, a fine powder of frozen leaves (100 mg) was taken in 1 ml Phosphate buffer solution (PBS) (pH 7.0). The mixture was centrifuged at 5°C and 8,064 rcf for 5 min. Extraction of enzyme was performed in 3 ml of 100 mM PBS (pH 7.0 and swirled for 15 min at 5 C and 8064 rcf). To 0.1 ml of plant extract, 3 ml PBS (100 mM), 0.05 ml guaiacol (20 mM), and 0.03 ml H_2_O_2_ (12.3 mM or 0.04%) were added and vortexed. Optical density was monitored at 436 nm, and change in the absorption over time was recorded. The activity of POD was calculated as follows:


(3) $$\begin{array}{r} Enzyme\;activity=\left(\frac{500}{\text{Δ}t}\right)\times \left(\frac{1}{1000}\right)\times \left(\frac{TV}{VU}\right)\times \left(\frac{1}{\left(f\;wt\right)}\right) \end{array}$$

where Δt = change in time; TV = total prepared volume and VU = volume of the sample used; f wt = fresh weight (g).

#### Determination of phytohormones in seedling's leaves

To extract phytohormones from seedlings, 100 mg of frozen leaf sample were ground to a fine powder in liquid nitrogen and taken in cold 1.5 ml eppendorf tube. The leaf powder was mixed with a 750 μl cold solution containing methanol:water:acetic acid (80:19:1, v/v/v). After vigorously shaken in the dark for 16 h at 4°C, the homogenate was centrifuged at 13,000 rpm for 15 min at 4°C. The pellet was re-extracted in 400 μl of extraction solution and centrifuged. The aliquots of supernatant were pooled and passed through syringe filter fitted with nylon filter (13-mm diameter and pore size 0.22 μm; Nylon 66; Jinteng Experiment Equipment Co., Ltd, Tianjing, China). The filtrate was evaporated under reduced pressure and re-dissolved in 200 μl methanol. The extract was used to quantify different phytohormones as described for fungal culture filtrate.

#### Reactive oxygen species (ROS) assay

##### ROS visualization via DAB

Total ROS was detected through DAB (Diaminobenzidine) staining technique (Keshavarz Tohid and Taheri, 2015). For this purpose, leaves of the seedling were put in Petri plates containing DAB stain solution (2 ml). The plates were kept at room temperature and 100 rpm for 4 h. The dyed leaves were then bleached with ethanol: acetic acid: glycerol (3:1:1) for removing chlorophyll. Leaves were then visually and microscopically observed for DAB staining, an indication of ROS.

Levels of H_2_O_2_ and O^2^ were assessed by studying the oxidation of fluorescent probes including 20,70-dichlorodihydrofluorescein diacetate (H_2_DCFDA, Molecular Probes, Leiden, The Netherlands) and dihydroethidium (DHE, Molecular Probes, Leiden, The Netherlands), respectively. The plant leaves (100 mg) were crushed in liquid nitrogen, and the powder was then added in 1 ml of 10 mM Tris–HCl (pH 7.2) taken in a 2 ml tube. Centrifugation of the sample was carried out at 12,000 rpm for 20 min at 4°C. The supernatant (100 μl) was added to a fresh eppendrof containing 900 μl of 10 mM Tris–HCl (pH 7.2). Aliquots of supernatants were mixed with 10 μl of 1 mM DHE or H2DCFDA prepared in PBS. After setting the excitation and emission wavelengths, the intensity of fluorescence was recorded every 15 min for 90 min (Alexandre et al., 2006).

#### Root colonization

Root colonization by the endophytic fungus was observed by staining roots with lactophenol cotton blue dye (Mehmood et al., 2019). Transverse sections of the roots were cut and observed under light microscope.

### Statistical analysis

The data obtained was analyzed statistically for significance by using SPSS for Windows (ver 21.0). Means of the groups (Two treatments) were compared using *t*-test at *p* = 0.5.

## Results

### Isolation, characterization, and screening on of endophytes

Eight different endophytes were isolated from wheat plant (Supplementary Table 1). Colonization frequencies of these strains in different parts of wheat plant ranged between 60 and 100%. All the isolated fungi were initially screened on wheat seedlings and *A. fatua* for their growth promoting or inhibiting activities, respectively. Based on initial screening, the strain GW was picked up for further study because of its excellent growth promoting and weedicide effect against wheat and *A. fatua*, respectively (Supplementary Table 1).

**Figure 1:**
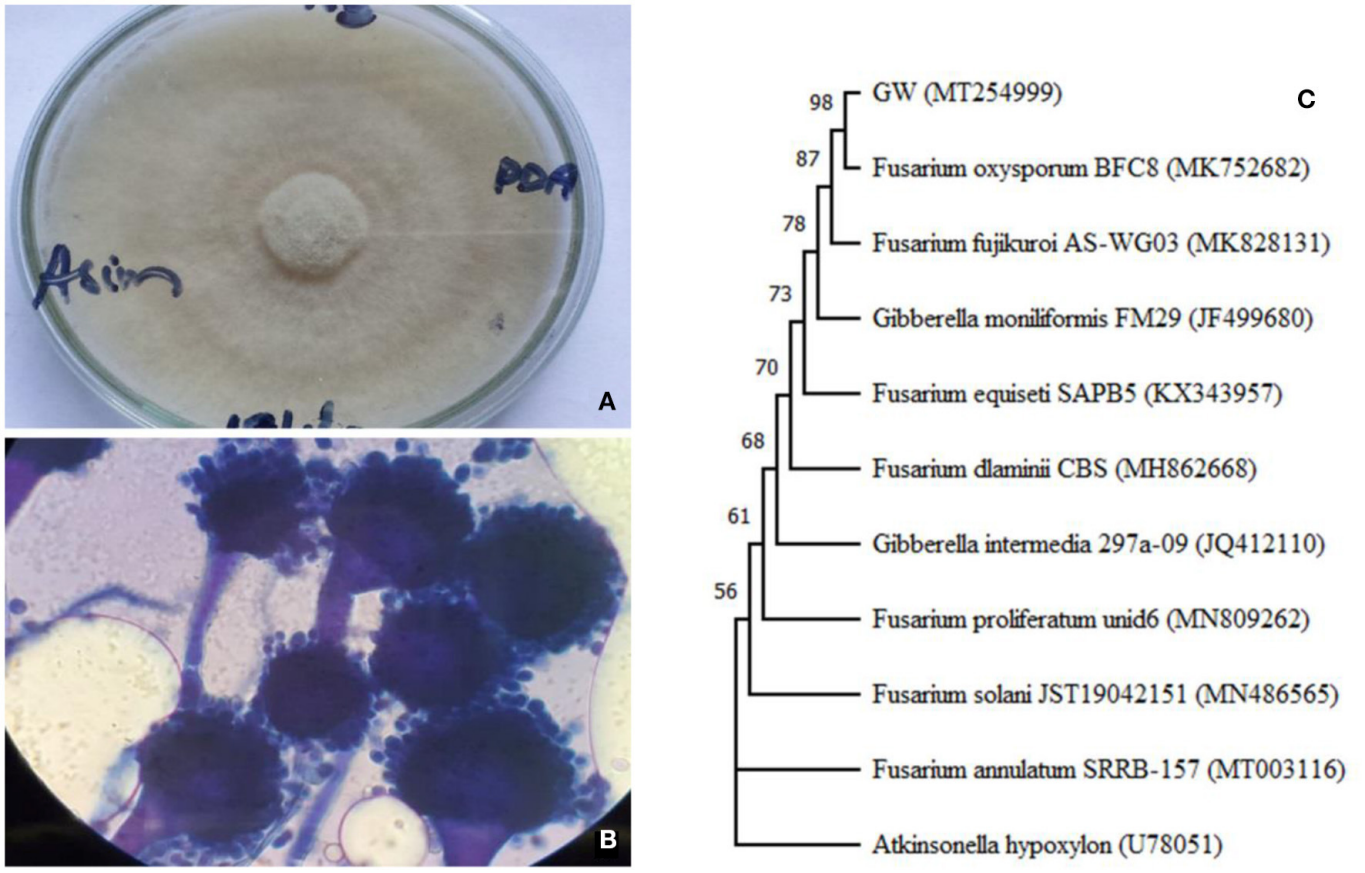
Colony morphology **(A)** fungal morphology, **(B)** light microscopy showing conidiophores at 500× magnification, and **(C)** phylogenetic relationship of ITS sequence of *Fusarium oxysporum* GW isolated during the current study. The neighbor-joining tree was made including the ITS sequence of *Atkinsonella hypoxylon* (U78051) as an out-group.

Initially, the isolate was characterized by observing its colony on PDA and mycelium under light microscope. The colonial characteristics of the isolates were: circular, velvety, and mottled type of growth with gray and yellow-brown colored colony on PDA medium (Figure 1A). The colonies had entire margins and umbonate elevation. Microscopic observation of 7 days old mycelium stained with lactophenol cotton blue showed the presence of conidiophore, spores, metulae, and phialides structures in isolates (Figure 1B). These features match with *Fusarium oxysporum*. Further confirmation was done using phylogenetic relationship and ITS sequence homology which indicated that the isolate was *F. oxysporum* (Figure 1C). The sequence (accession No. MT254999) is available on NCBI GenBank.

Of the 8 isolates, GW was selected for further study based on its ability to biofertilize wheat and inhibit weed (*A. fatua*) growth. The strain was identified as *F. oxysporum* based on its morphological and molecular features (ITS sequencing).

### Growth assay of wheat and *A. fatua*

**Figure 2:**
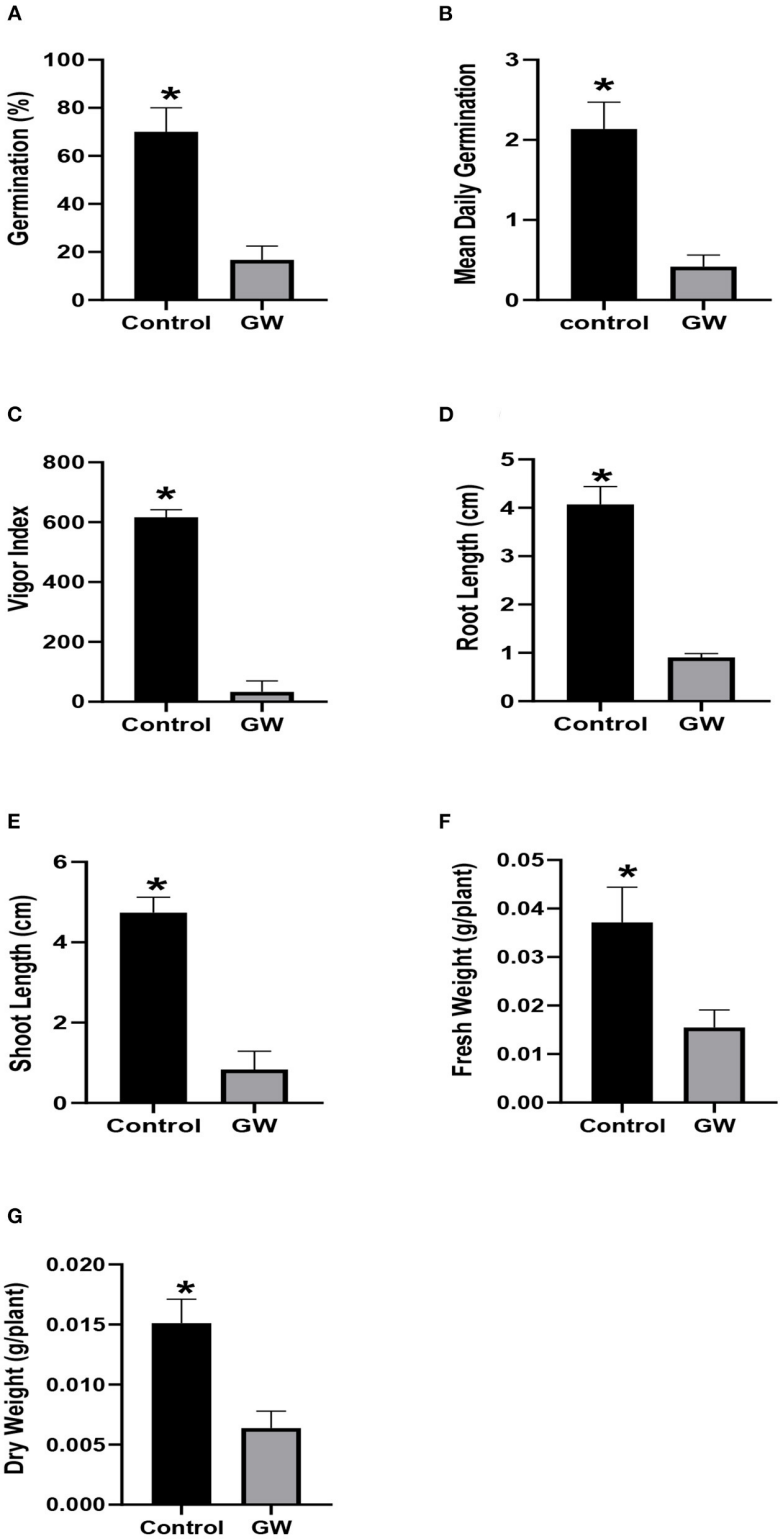
Effect of FCF from endophytic fungus *F. oxysporum* GW on the germination **(A)**, mean daily germination **(B)**, Vigor index **(C)**, root length **(D)**, shoot length **(E)**, fresh weight **(F)**, and dry weight **(G)** of *Avena fatua* seedlings for 1 week after germination. Bars show the mean of three replicates with standard deviation. Bars labeled with * are significantly higher (*t*-test; *p* < 0.05).

Culture filtrate (FCF) of the selected fungus was assessed for effect on the growth of wheat and *A. fatua* seedlings grown for 1 week. In *A. fatua* seedlings grown in Petri plates containing GW FCF, % seed germination and mean daily germination (MDG) were reduced to 20.81 and 17.1% of the distilled water control seedlings (Figures 2A,B). The effect of FCF was more pronounced on vigor index, reducing it to only 5.4% of the control (Figure 2C). Shoot and root growth was also inhibited significantly in FCF-treated seedlings (Figures 2D,E). The Weedicide effect of FCF was also noticed on *Avena* biomass which was reduced down to 59.4% even lower than the control (Figures 2F,G).

**Figure 3:**
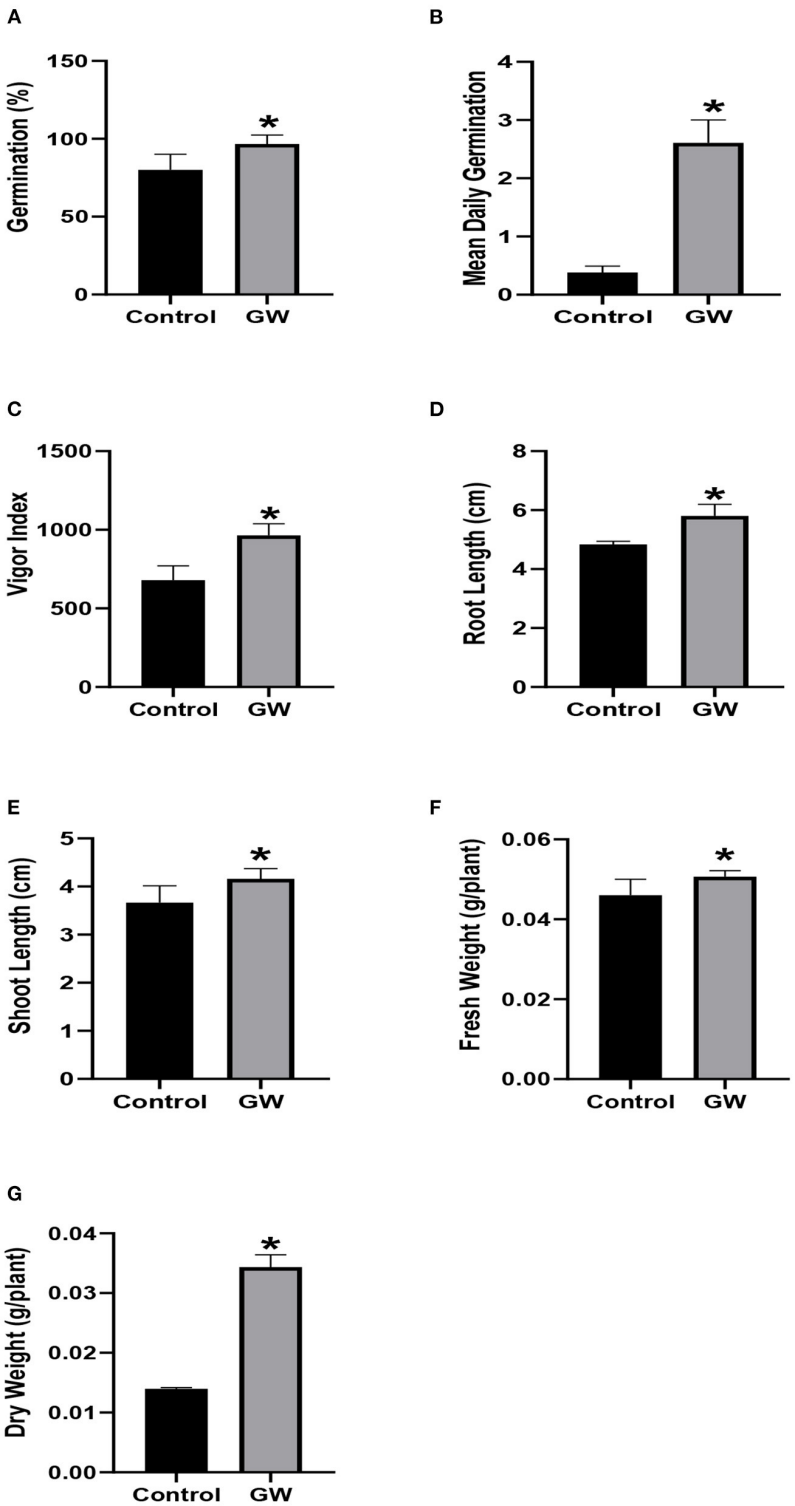
Effect of FCF from endophytic fungus *F. oxysporum* GW on the germination **(A)**, mean daily germination **(B)**, Vigor index **(C)**, root length **(D)**, shoot length **(E)**, fresh weight **(F)**, and dry weight **(G)** of wheat seedlings for 1 week after germination. Bars show the mean of three replicates with standard deviation. Bars labeled with * are significantly higher (*t*-test; *p* < 0.05).

Contrary to its inhibitory effect on *Avena*, the growth of wheat seedlings was significantly improved by GW (Figure 3A). Seed germination, mean daily germination, and vigor index were significantly greater in GW-associated seedlings than the control. Similarly, root length, shoot length, fresh weight, and dry weight were also enhanced by 16.59, 11.94, 9, and 62%, respectively, in GW-associated seedlings in comparison to the control (Figure 3).

Culture filtrate had the ability to promote growth of wheat and inhibit growth of *A. fatua*.

### Identification of compounds in FCF fractions and their effect

The FCF was fractionated using preparative HPLC reverse phase C18 column. We collected three different fractions which were labeled as fractions A, B, and C (Supplementary Figure 1). These fractions were eluted at 1.17, 1.87, and 8.4 min, respectively. The fractions were then bioassayed on wheat and *Avena* seeds (Supplementary Table 2). In comparison to control *Avena* seeds, fraction A restricted seed germination, mean daily germination, and vigor index to 10, 25.8, and 6.04% of the control, respectively. This fraction also inhibited other growth parameters of the weed including root length, shoot length, fresh weight, and dry weight (Supplementary Table 2). The remaining two fractions completely abolished the germination of the seeds.

The fractions were analyzed through LC-ESI-MS/MS to identify the compounds present. Fraction A had four different flavonoids including Quercetagetin, Isovitexin, Calycosin, and Dihydroxy-dimethoxyiso?avone (Table 1). Fraction B had three types of flavonoids, namely, Naringenin derivative, Vitexin, and Caffeoyl-D-glucose. Similarly, only two compounds, i.e., Cis-cafatric acid and p-hydroxy benzoic acid were identified in the fraction C.

**Table 1:** Identification of flavonoids, phenols and carbohydrates in HPLC Fractions of GW strain.

**Fraction**	**m/z**	**Ionization mode**	**Fragmentation (Ms^2^)**	**Compounds**	**References**
A	315	+ve	297, 283	Dihydroxy-dimethoxy-isoflavone	(Zhang et al., 2015)
A	283	–ve	283, 268, 265, 238, 213, 200, 184, 157, 152, 96	Calycosin	(Ye et al., 2012; Mehmood et al., 2019)
A	431	–ve	431, 341, 311, 283, 341	Isovitexin	(Ye et al., 2012)
B	325	–ve	325, 307, 289, 271, 263, 185, 169	Naringenin derivative	(Chen et al., 2016)
B	431	–ve	431, 341, 311, 283, 269	Vitexin	(Chen et al., 2016)
B	339	–ve	275, 269, 179, 161, 143	Caffeoyl-D-glucose	(Shakya and Navarre, 2006)
C	311	–ve	296, 247	Cis-cafatric acid	(Lambert et al., 2015)
C	137	–ve	137, 119, 93	p-hydroxybenzoic acid	(Arranz et al., 2010)

*(m/z), mass to charge ratio*.

Fungal culture filtrate was fractionated into three fractions, and all of the fractions had an inhibitory effect on weed. The fractions had a number of flavonoids, majority of which are known for weed control potential.

### Determination of secondary metabolites in fungal culture filtrate

**Figure 4:**
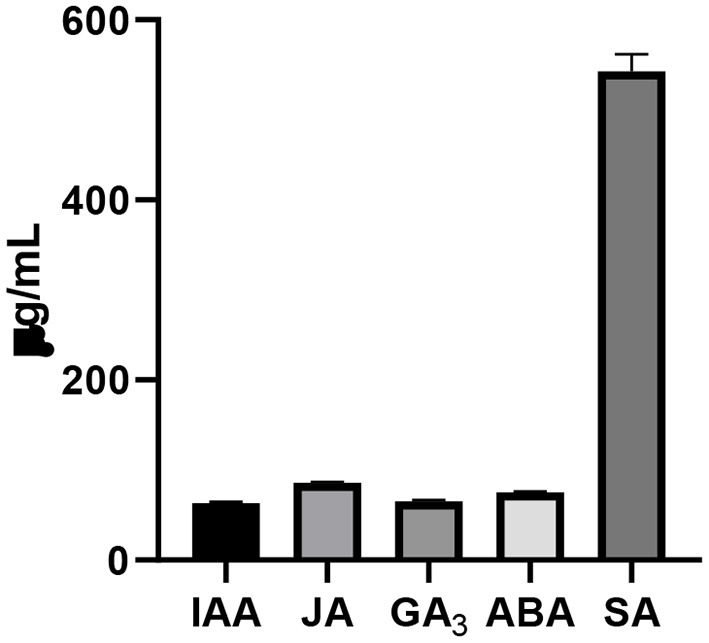
Determination of different phytohormones including IAA, JA, GA3, ABA, and SA in the culture filtrate of the isolated endophytic *F. oxysproum* GW grown in Capek medium for 5 days. Bars show the mean of three replicates with standard deviation.

The endophyte produced significant quantities of total phenols (189 μg/ml) and flavonoids (304 μg/ml), which are shown in Supplementary Figure 2. The fungus released various phytohormones including IAA, JA, GA3, ABA, and SA (Figure 4). The concentration of SA was the highest among different phytohormones determined in the culture filtrate.

### Weedicide potential of GW against *Avena fatua*

**Figure 5:**
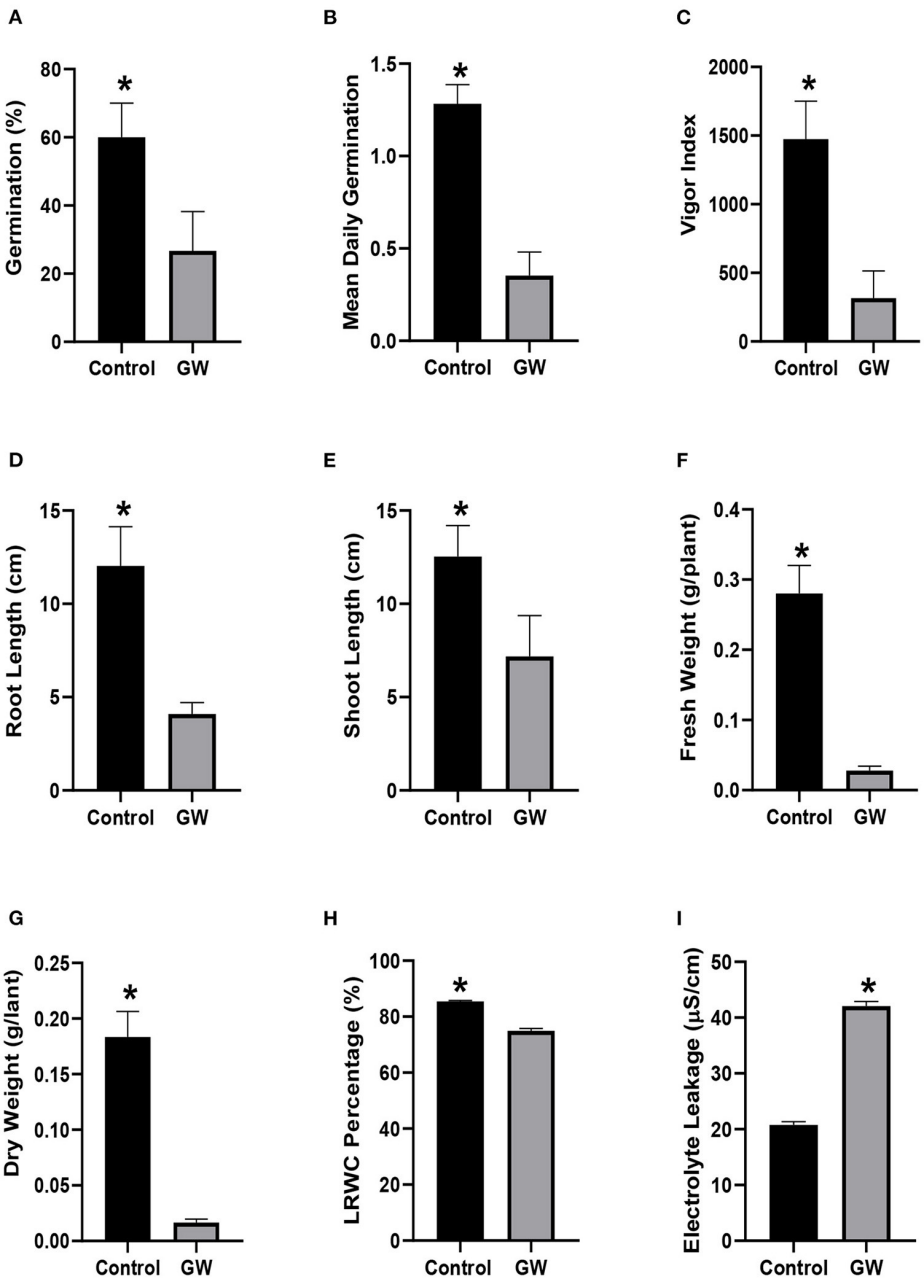
Weedicide potential of GW against *Avena fatua* **(A)** germination percentage, **(B)** mean daily germination, **(C)** vigor index, **(D)** root length, **(E)** shoot length, **(F)** fresh weight, **(G)** dry weight, **(H)** LRWC, and **(I)** EC. Bars show the mean of three replicates with standard deviation. Bars labeled with * are significantly higher (*t*-test; *p* < 0.05).

Soil containing biomass of GW had a significant impact on the growth of wheat and weed seedlings (Supplementary Figure 3). Application of GW in soil not only reduced the percent germination of *A. fatua* seedlings but also delayed their germination by significantly reducing the mean daily germination rate (Figures 5A,B). Another indication of the weedicide potential of the isolate was the lower vigor index of seedlings grown in GW inoculated soil (Figure 5C). The seedlings were hit hard by our isolated endophyte as indicated by significantly lower root and shoot growth than the control seedlings (Figures 5D,E). Seedlings inoculated with GW also had significantly lower biomass than the control seedlings (Figures 5F,G). Similarly, GW inoculated *A. fatua* seedlings had higer electrolyte leakage and lower relative water content than the non inoculated control (Figures 5H,I).

#### GW modulated physiology and biochemistry of *A. fatua* seedlings

**Figure 6:**
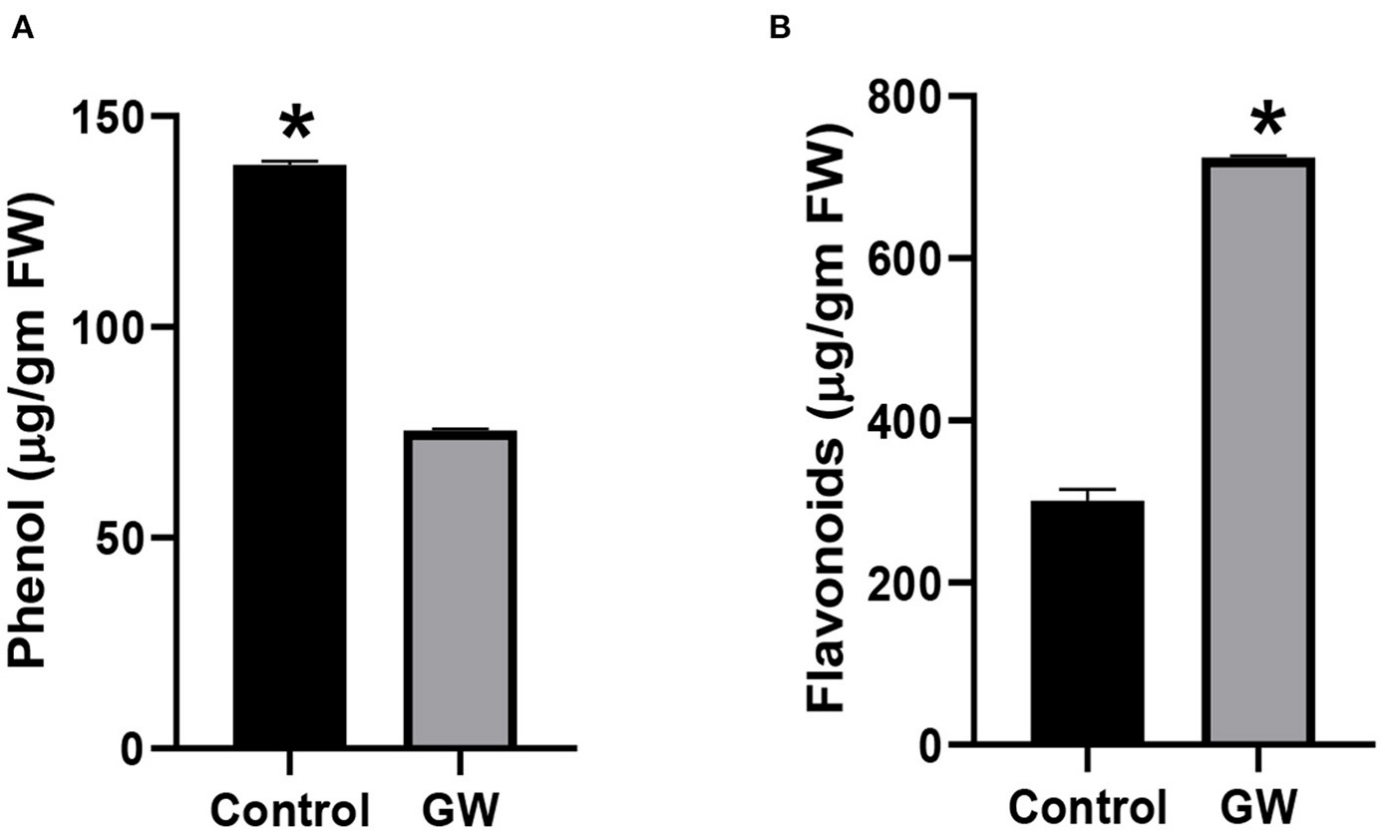
Determination of **(A)** phenol and **(B)** flavonoid in the leaves of *A. fatua* seedlings grown in the presence and absence of endophytic fungus *F. oxysporum* GW. Bars show the mean of three replicates with standard deviation. Bars labeled with * are significantly higher (*t*-test; *p* < 0.05).

Different secondary metabolites in the leaf of *A. fatua* were monitored in order to score the effect of GW on seedlings physiology and biochemistry. Seedlings grown on soil containing GW strain had a significantly lower amount of total phenols in comparison to control seedlings (Figure 6A). Interestingly, flavonoid concentration was significantly higher in GW-associated seedlings than the control (Figure 6C). Leaf relative water contents of *A. fatua* seedlings inoculated with GW were lower than the control seedlings indicating a damaged cell membrane under the influence of GW (Figure 6).

**Figure 7:**
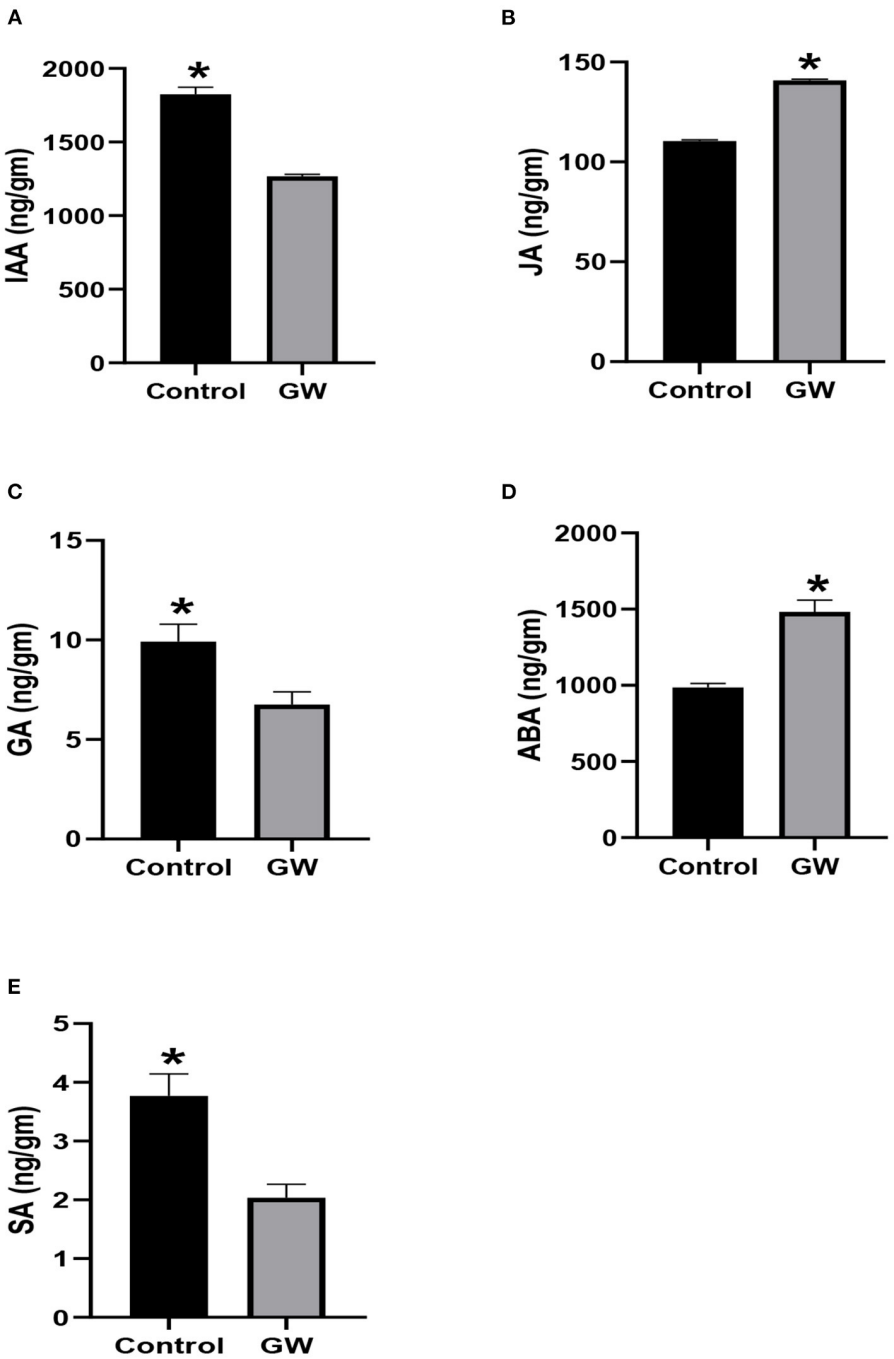
Determination of different phytohormones **(A)** IAA, **(B)** JA, **(C)** GA, **(D)** ABA, and **(E)** SA in *Avena fatua* inoculated with the endophytic fungus *Fusarium oxysporum* GW. The seedlings were grown for 21 days inautoclaved soil inoculated with fungal biomass. Bars show the mean of three replicates with standard deviation. Bars labeled with * are significantly higher (*t*-test; *p* < 0.05).

The fungus Gw significantly influenced the endogenous phytohormones pool of its host *A. fatua*. The endogenous level of IAA, GA3, and SA was significantly lower in GW-associated *A. fatua* seedlings than the control (Figure 7). Contrary to this, JA and ABA levels were greater in the GW-infected seedlings.

**Figure 8:**
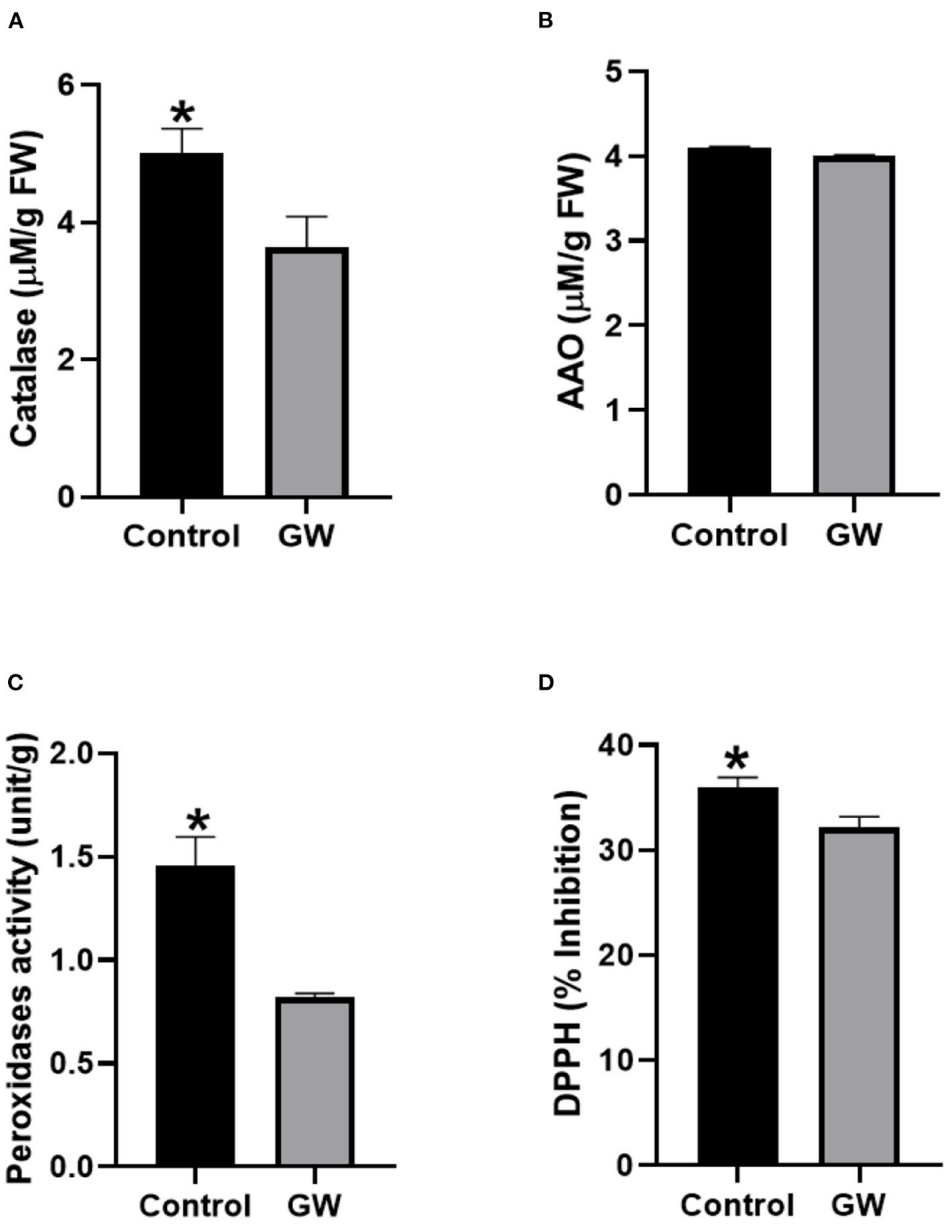
Activities of antioxidant enzymes including **(A)** catalase, **(B)** AAO, **(C)** PO, and **(D)** percent inhibition of DPPH (ROS scavenging assay) in the leaf extract of *A. fatua* seedlings. Bars show the mean of three replicates with standard deviation. Bars labeled with * are significantly higher (*t*-test; *p* < 0.05).

Different antioxidant assays were performed on *Avena* leaf extract to assess the nature of oxidative stress management in seedlings colonized by the weedicide GW. Interestingly, the activity of catalase, ascorbic acid oxidase, and peroxidase was significantly lower in GW colonized seedlings than the control (Figures 8A–C). Similarly, DPPH scavenging potential of the GW-associated seedling was lower than the control seedlings (Figure 8D).

### Wheat growth promotion by GW

**Figure 9:**
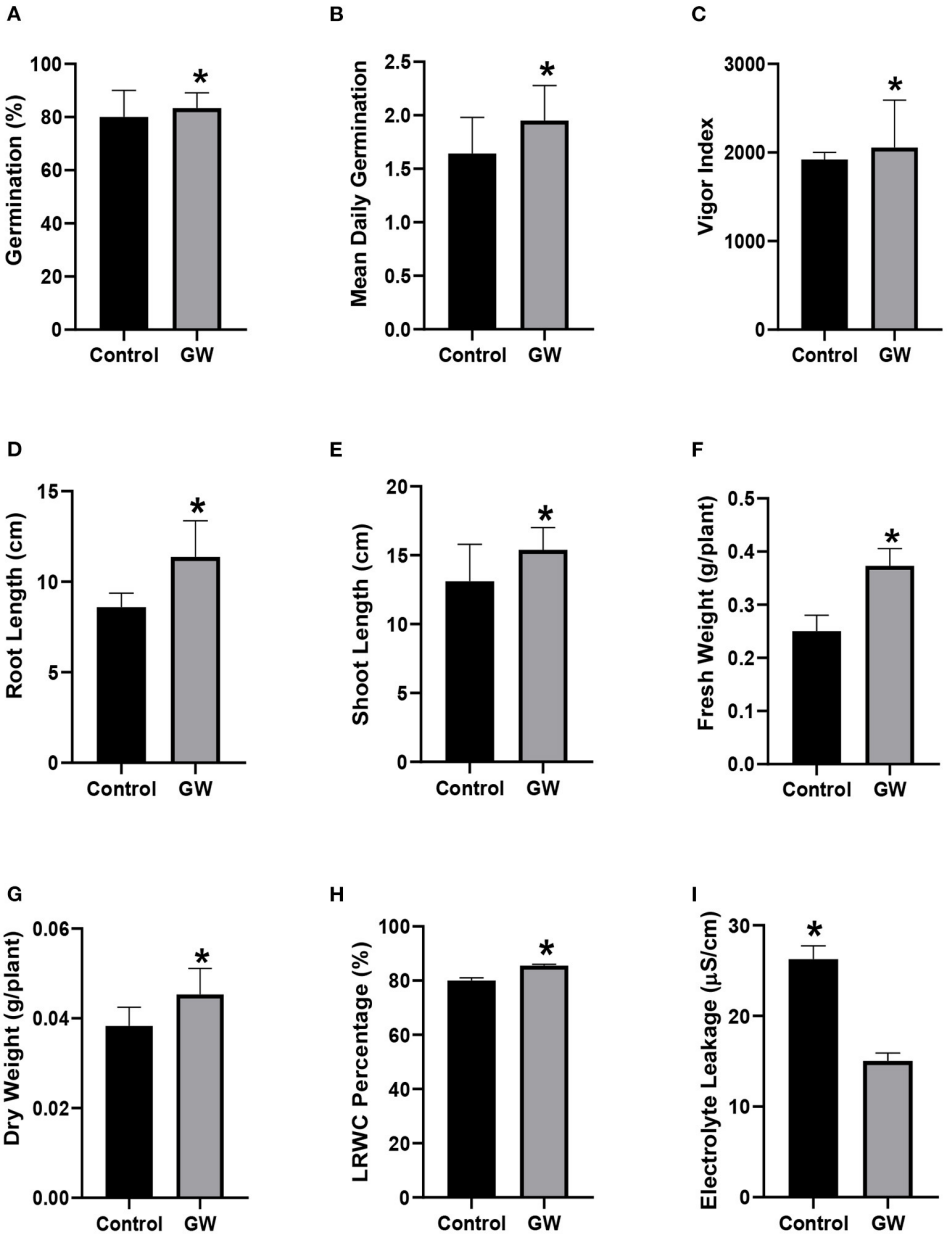
Wheat biofertilization by *F. oxysporum* GW **(A)** germination percentage, **(B)** mean daily germination, **(C)** vigor index, **(D)** root length, **(E)** shoot length, **(F)** fresh weight, **(G)** dry weight, **(H)** LRWC, and **(I)** EC. Bars show the mean of three replicates with standard deviation. Bars labeled with * are significantly higher (*t*-test; *p* < 0.05).

Fungal biomass was collected and mixed in soil which was then used to grow wheat seedlings for 21 days. In the presence of fungal endophyte GW, seed germination and mean daily germination rate were significantly improved than the control seedlings (Figures 9A,B). Endophyte-associated seedlings also had higher Vigor index, roots and shoot growth than the non-endophyte control seedlings (Figures 9C–E). Fresh and dry biomass of the GW colonized seedlings were 33 and 15.5% greater than the control, respectively (Figures 9F,G). Leaf relative water content of GW colonized seedlings was much improved over control seedlings (Figure 9H). Electrolyte leakage was significantly lower in endophyte associated seedlings than the control (Figure 9I).

#### GW-modulated physiology and biochemistry of wheat seedlings

**Figure 10:**
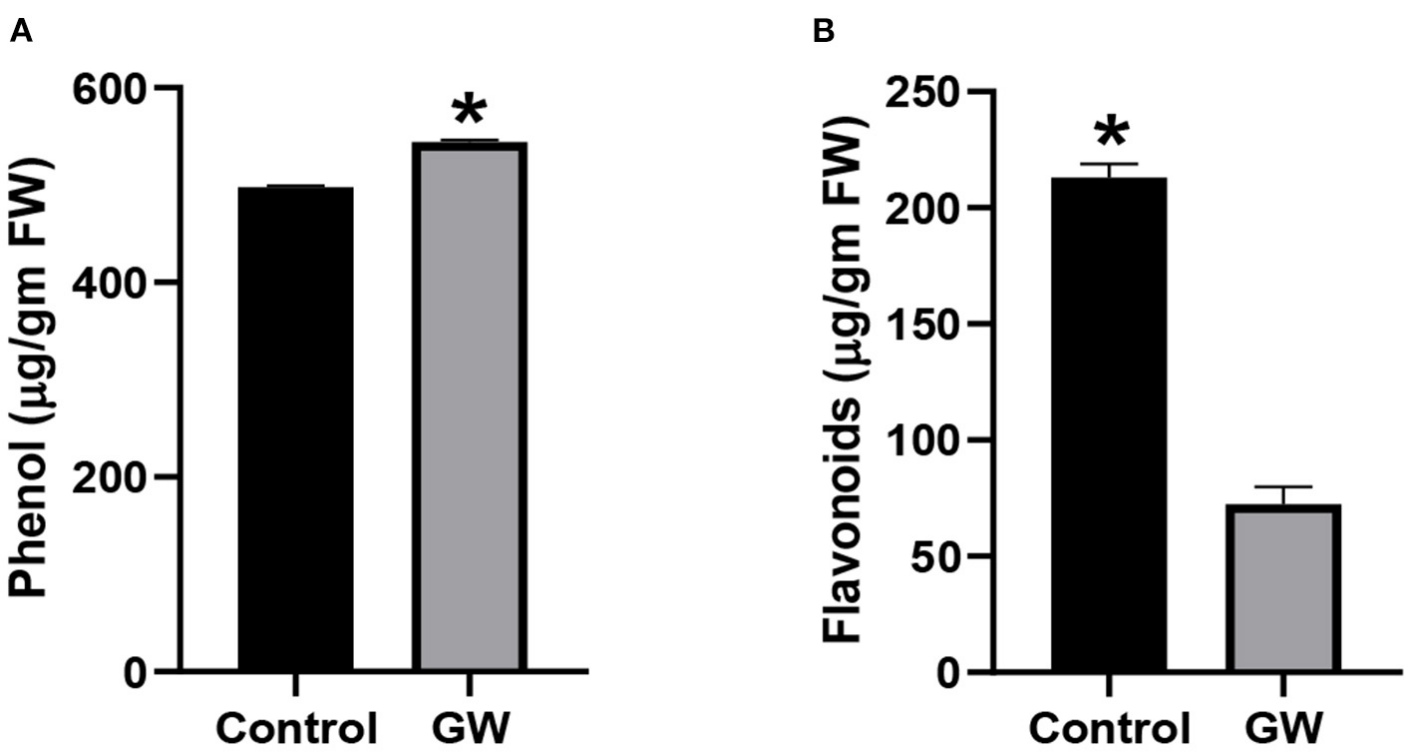
Determination of **(A)** phenol and **(B)** flavonoids in the leaves of wheat seedlings grown in the presence and absence of *F. oxysporum* GW. Bars show the mean of three replicates with standard deviation. Bars labeled with * are significantly higher (*t*-test; *p* < 0.05).

The GW-associated seedlings were also screened for alteration in the production of secondary metabolites including flavonoids and phenols. Production of total phenols was promoted in GW colonized seedlings by 8.55% than the control (Figure 10A). However, GW-associated seedlings were characterized by a significant reduction in flavonoids than the control seedlings (Figure 10B).

**Figure 11:**
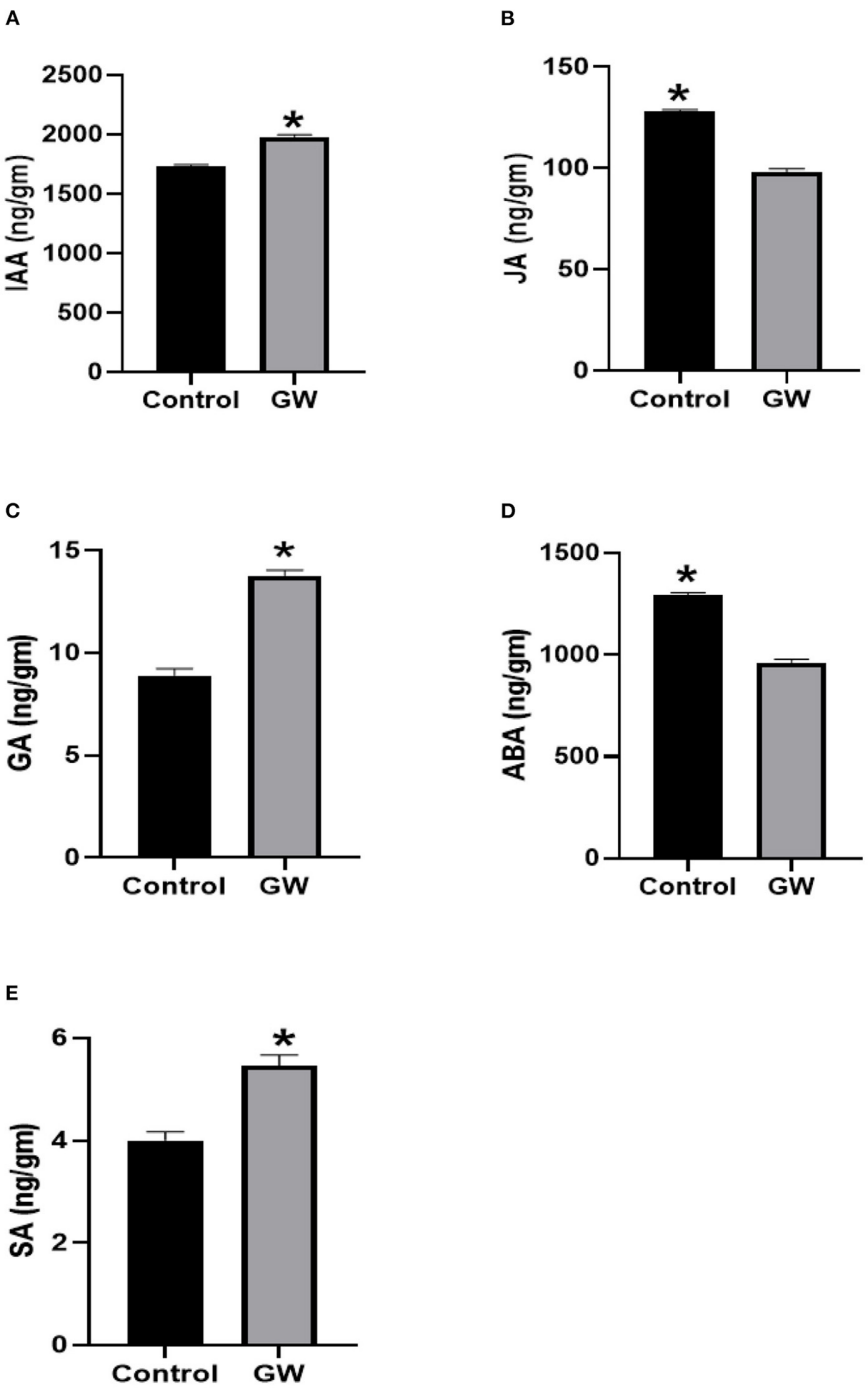
Determination of different phytohormones **(A)** IAA, **(B)** JA, **(C)** GA, **(D)** ABA, and **(E)** SA in wheat seedlings inoculated with the endophytic fungus *Fusarium oxysporum* GW. Wheat seedlings were grown for 21 days in autoclaved soil inoculated with fungal biomass. Bars shows the mean of three replicates with standard deviation. Bars labeled with * are significantly higher (*t*-test; *p* < 0.05).

In GW-colonized wheat seedlings, quantities of IAA, GA3, and SA were greater when compared with the control. However, the levels of JA and ABA were significantly reduced in seedlings colonized by the endophyte GW (Figure 11).

**Figure 12:**
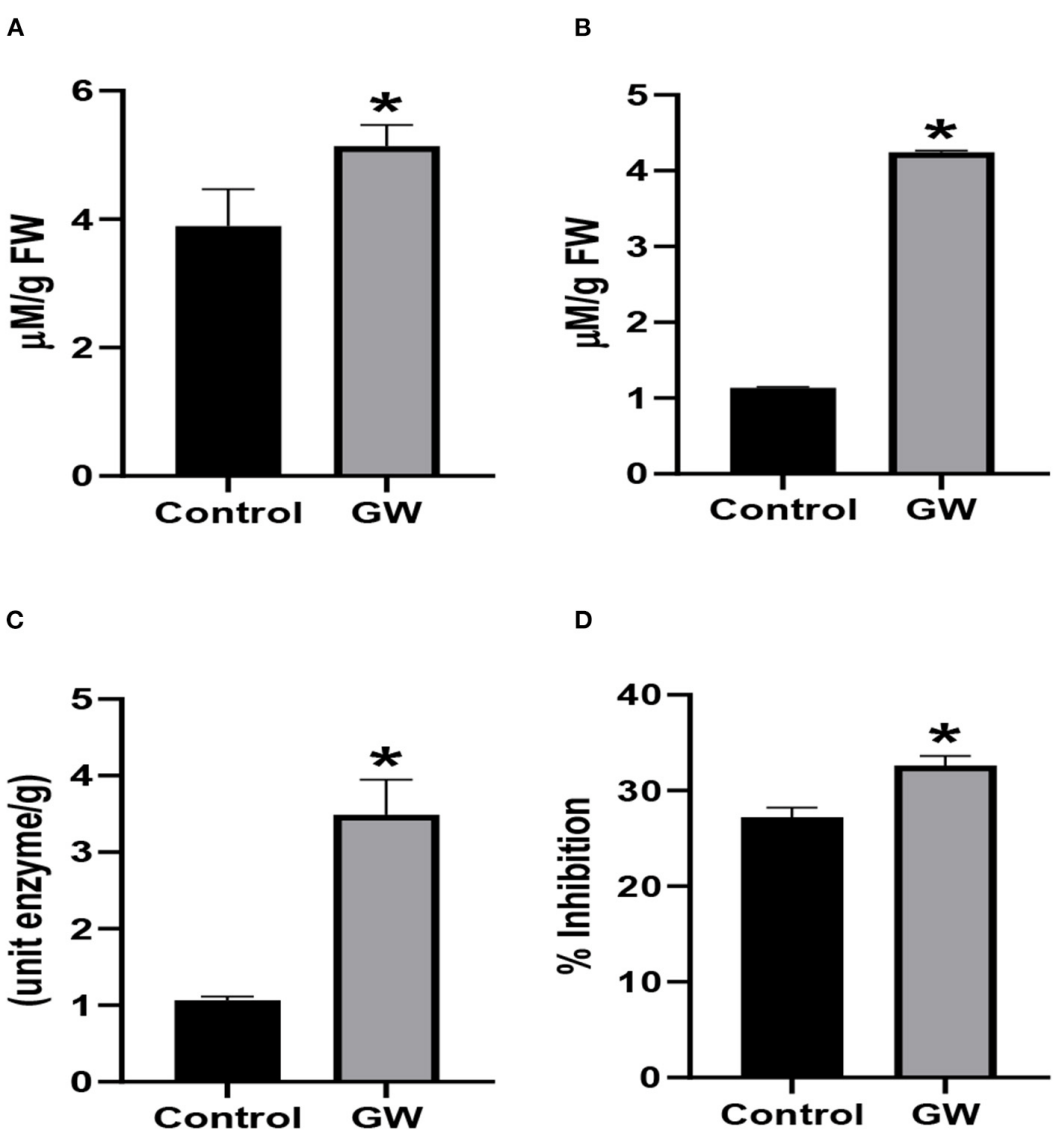
Effect of *Fusarium oxysporum* GW on the activities of antioxidant enzymes **(A)** catalase, **(B)** AAO, **(C)** peroxidase, and **(D)** percent inhibition of DPPH (ROS scavenging activity) in wheat seedlings. Assays were performed on the leaf extract of wheat seedlings grown for 21 days under control conditions. Bars labeled with * are significantly higher (*t*-test; *p* < 0.05).

Antioxidant system was strengthened in seedlings inoculated with GW. The activity of AAO and PO was enhanced by 73.28 and 96.9 % in GW colonized seedlings, respectively, in comparison to the control (Figures 12B,C). Also, the DPPH scavenging activity was significantly higher in GW-associated seedlings than the control (Figure 12D). However, the activity of catalase was not different in endophyte and non-endophyte seedlings (Figure 12A). Higher leaf relative contents of inoculated with GW than the control signifying virtuous infirmity of cell membrane wheat seedlings. Catalase, ascorbic oxidase, peroxidase, and DPPH activities show an inordinate increase with GW treatment in wheat seedlings (Figure 12D).

### Accumulation of ROS species in the host plants

Colonization of wheat by GW strain had no influence on the accumulation of ROS in the leaves as indicated by completely clear leaves post DAB staining. Contrary to this, colonization of *Avena* by GW-induced accumulation significantly as indicated by intense DAB stain in the leaves, in comparison to the control leaves (Supplementary Figure 4).

**Figure 13:**
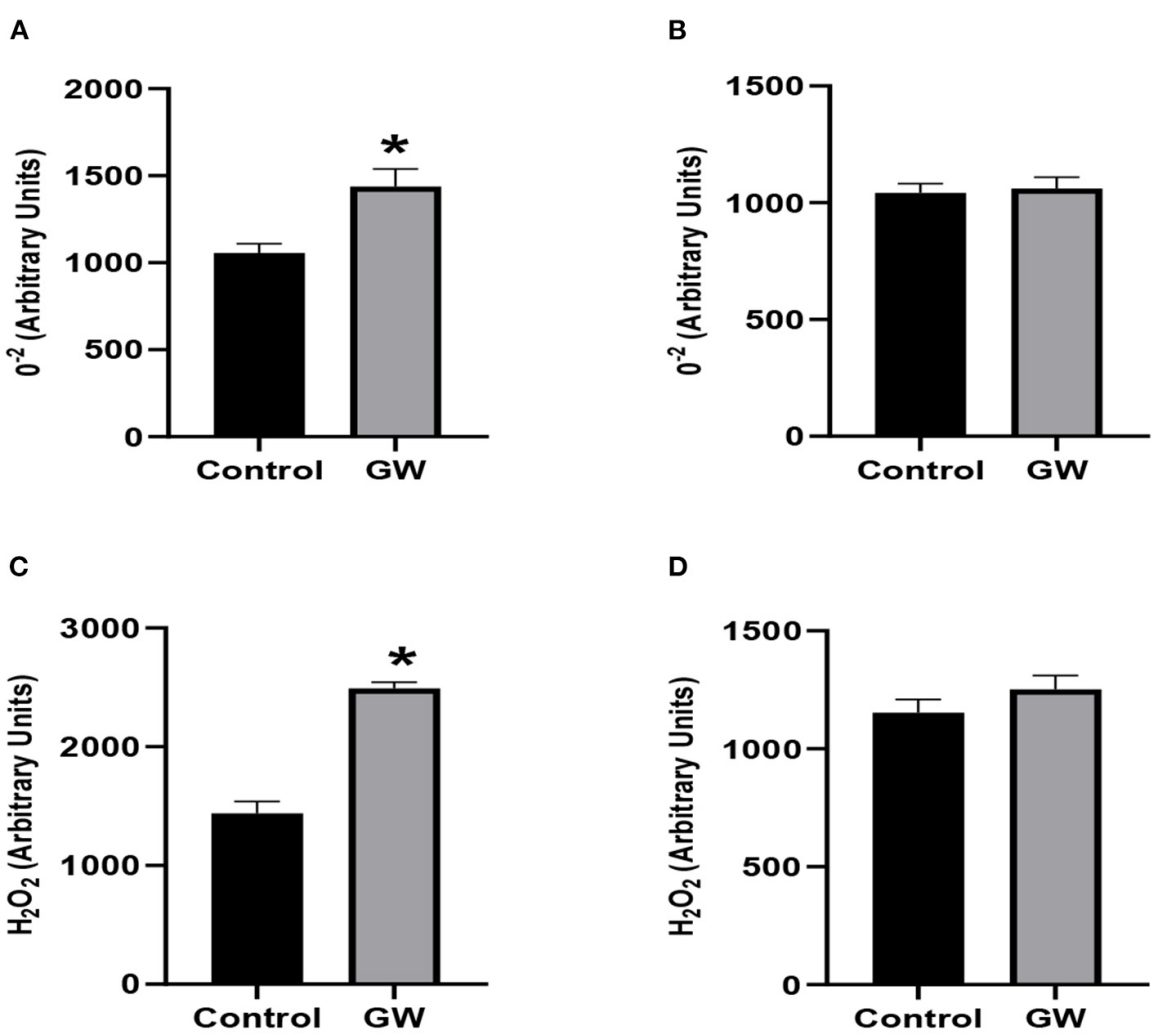
Effect of endophytic fungus *Fusarium oxysporum* GW on the level of H_2_O_2_ and O^2−^ in the leaves of *A. fatua* weed **(A,C)** and wheat **(B,D)**. Bars labeled with * are significantly higher (*t*-test; *p* < 0.05).

Significantly enhanced accumulation of reactive oxygen species was observed in the leaves of *A. fatua* seedlings inoculated with the GW isolate (Figures 13A,C). No significant change in the ROS accumulation was observed in wheat leaves between control and endophyte-associated seedlings. To resolve the leaves ROS species, fluorimetric analysis of O^2−^ and H_2_O_2_ was carried out in *A. fatua* and wheat seedlings. A sharp increase in the accumulation of selected ROS species was obvious in endophyte-associated *A. fatua* leaves (Figures 13B,D). Contrary to this, ROS species accumulation declined in wheat seedlings as a result of GW inoculation.

### Host root colonization

To confirm root colonization by the isolated endophyte, lactophenol cotton blue stain was applied on the root segments, and TS was observed under light microscopy. The fungus was able to colonize both the host equally well (Supplementary Figure 5). Interestingly, the fungus colonized all the root tissues including epidermis, cortex, and vascular region. The pith region was not colonized by the fungus in the roots of both the host seedlings.

## Discussion

During the current study, we isolated eight different endophytic fungi from wheat, most of which were capable to promote growth seedlings. However, the isolate GW was the only one to significantly inhibit the growth of an important weed, *A. fatua*, which is a common weed competing with wheat (Mustafa et al., 2019). Based on its ability to promote the growth of wheat and check the growth of *A. fatua*, the strain was studied in detail and identified as *F. oxysporum* GW. The fungus, *F. oxysporum*, has previously been identified as both endophyte and plant pathogen (Cui et al., 2012; Nirmaladevi et al., 2016); (Caicedo et al., 2019). Contrasting effect of this fungus on wheat and *A. fatua* makes it an ideal weedicide, at least under lab conditions.

The selected endophyte was very effective in controlling weed at different stages of life cycle including seed germination and seedling stages. Seed germination was not only cut down to below 20% but also significantly delayed as to <1 seed germinated per day. Also, seedlings vigor index was significantly lower in GW inoculated seedlings. Hence, GW works both as pre and post emergence weedicide against *A. fatua*. This annual weed is reproduced by seeds that remain viable for up to 8 years in the soil. Hence, control on seed germination would be an important strategy to control this weed. Previous reports have shown that fungal endophytes produce metabolites that are toxic to certain weeds but safe for crop plants (Suryanarayanan, 2019).

To find out potential herbicide in the culture of *F. oxysporum* GW, its culture filtrate was fractionated and each fraction was tested on *A. fatua*. Bioactive fractions had a number of polyphenols including quercetagetin, isovitexin, calycosin (Lou et al., 2016), dihydroxy-dimethoxyisoflavone, naringenine (Kaab et al., 2020), vitaxin, caffeoyl-D-glucose, cis-cafatric acid, and p-dydroxy benzoic acid. Isovitexin has previously been shown to exhibit allelopathic and herbicidal potential against several weeds including *Amaranthus retroflexus* L., *Portulaca oleracea* L., *Chenopodium album* L., and *Abutilon theophrasti* Med. (Boselli et al., 2021). Calycosin has also been shown to possess strong weedicide activities and some plants including red clover use these flavonoids to suppress the growth and germination of the competing weeds (Lou et al., 2016). Weedicide potential of quercetagetin and dihydroxy-dimethoxyisoflavone against important weeds of Convolvulus species has been documented in Egypt (Balah, 2012). The presence of these potent polyphenols in the culture of *F. oxysporum* GW was a strong signal of their weedicide potential. Hence, the strain was used in pot experiment for control of the selected weed. However, before going to pot experiment, some of these compounds were purchased and tested against *A. fatua* in lab experiment to confirm that these polyphenols were effective in pure form and in different combination. The compounds including quercetagetin, calycosin (Lou et al., 2016), and naringenine (Kaab et al., 2020) were weakly weedicides against *A. fatua*. In binary combination, their activities enhanced synergistically. When all the three polyphenols were applied in combination, their potency *A. fatua* increased.

To look into further details of weedicide potential of the endophytic fungus, *A. fatua* inoculated with the fungus was assessed for physiological and biochemical markers. Despite of the fact that the endophytic fungus released IAA, *A. fatua* seedlings colonized by this fungus had significantly reduced IAA level, which seems to be due to growth inhibition induced by the polyphenols secreted by the fungus. Total phenols were also significantly lower in endophyte-associated weed seedlings. Phenols are of vital importance in defending plants against biotic stress and a sharp host response to re-program its phenol production decide the fate of pathogenicity (Jan et al., 2019). Evidence shows that the phenol profile of the host changes upon encountering infection and the host could start accumulating different phenols than the uninfected counterpart. The overall concentration of phenols might not always increase in the host and an inadequate response may lead to a reduced accumulation of total phenols (Aybeke, 2020). During the current study, reduced phenols concentration in the infected weed seedlings was linked with reduced radical scavenging activity leaving the weed unprotected against the uncontrolled production of ROS. Enzymatic arm of the antioxidant system was also inadequate for countering the pathogen. Failure of the antioxidant system leads to severe impairment of growth in the infected weed.

An important finding of the current study was the differential response of wheat and weed seedlings to the same fungus *F. oxysporum* GW. *A. fatua* seedlings had a lower concentration of different phytohormones including IAA, GA3, JA, and SA than the control seedlings. The response of ABA was opposite to other phytohormones. Previous studies have shown that infection by *F. oxysporum* causes phytohormones disorder in a weed, *Orobanche* spp. (Aybeke, 2017). However, instead of SA-dependent defense response observed in *Orobanche, A. fatua* seedlings were left defenseless as SA level lowered significantly. Contrary to *A. fatua*, wheat seedlings had a higher concentration of phytohormones in response to GW inoculation. Exception was ABA which was lower in GW-associated seedlings than the control. One possible reason for phytohormones disorder in the weed seedlings may be ROS burst leaving the host with compromised growth and decline in phytohormones concentration. Due to ROS interplay with different phytohormones, the fate of plant response to environmental stresses is decided. The abiotic stress induces an increase in ROS accumulation which in turn regulate different plant hormones including GAs, IAA, ABA, and BRs (brassinosteroids). Conjugation and oxidative degradation of auxin are major ROS effects that lead to changes in auxin gradients (Mohanta et al., 2018). In the case of wheat, ROS accumulation was checked and kept at normal level, and the endophyte induced a higher accumulation of phytohormones, promoting seedlings growth.

Plants normally respond to intruder by elevated production of ROS which ensures to restrict the growth of the invading endophytes. However, ROS production is enhanced locally to trap the invading pathogens. In the case of symbiotic fungi, ROS generation and ROS accumulation are balanced by antioxidant enzymes keeping their levels near to normal (Lanfranco et al., 2005). However, during the current study, the endophyte efficiently colonized in root tissues, provoking systemic ROS accumulation in the leaves of weed seedlings. This indicates that the endophyte had a systemic effect on the physiology of *A. fatua*. An important fact is that unlike superoxide anions, H_2_O_2_ can easily cross biologically membrane and be transported far from its source site. H_2_O_2_ and O^2−^ are moderately reactive among ROS, but both can contribute in generating OH which is the most reactive ROS species. SOD and CAT inhibition block the formation of OH from H_2_O_2_ and O^2−^. Reduced CAT levels in endophytes associated *A. fatua* indicated lower conversion of H_2_O_2_ to OH radicals but resulted in higher accumulation of H_2_O_2_ which induced oxidative stress in the seedlings (Cembrowska-Lech, 2020). Also, superoxide anion accumulation was higher in endophytes-associated leaves of *A. fatua*. Taken together, these results indicate that GW induces systemic oxidative stress in *A. fatua* seedlings that contribute to growth inhibition and hypersensitive response.

Contrary to the inhibitory effect of polyphenols on weed, wheat growth was promoted through the induction of antioxidant system (Daripa et al., 2016). Previous studies have shown that the application of exogenous polyphenols boosts antioxidant enzymes of wheat plants and helps to alleviate the effect of abiotic stress (Lianbang et al., 2020). Accumulation of ROS species was maintained at a normal level in the leaves of endophyte-associated wheat seedlings. Because of the strengthened antioxidant system in such seedlings, the extra ROS that might have generated in response to the endophyte was scavenged to avoid any negative consequences.

Under low concentration of ROS, plants grow well due to improved signaling. However, in concentration above the optimum level, they become injurious to plant tissues which often leads to premature cell death. Luckily, plant antioxidant defense keeps ROS level under check in stressful conditions (Agrawal et al., 2003). Enzymes such as CAT and AAO can neutralize ROS directly or indirectly. In stressed plants, an excessive amount of H_2_O_2_ is degraded by CAT and AAO. Hence, their concentration in plants is linked with stress severity and susceptibility in plants (Waller et al., 2005). Previous findings have shown that the activities of these antioxidant enzymes vary in endophyte-associated plants. *Aspergillus japonicus* has been associated with a decrease in the activities of AAO and CAT of thermal stressed soybean and sunflower hosts (Waqas et al., 2012).

## Conclusion

The endophytic fungus *F. oxysporum* GW promotes the growth of wheat and inhibits the growth of the weed *A. fatua* by deferentially modulating their physiology and biochemistry. Inoculation of *A. fatua* by this fungus leads to pre and post germination impact which is reflected in lower seed germination, vigor index, and compromised growth of the host plant. Contrary to this response of wheat is totally different. The same is reflected in phytohormones profile of both plants. On the one hand, concentration of phytohormones including IAA, GA3, and SA was enhanced in wheat and reduced in *A. fatua* when these plants were inoculated with the fungus. On the other hand, ABA and JA showed the opposite trend. Similarly, in weed, ROS accumulation was enhanced and antioxidant system compromised under the influence of GW. Response of wheat was exactly opposite to the weed. Taken together, these results suggest that *F. oxysporum* GW has the potential to be tested as bioherbicide in wheat field for the control of *A. fatua*.

## Data availability statement

The datasets presented in this study can be found in online repositories. The names of the repository/repositories and accession number(s) can be found in the article/Supplementary material.

## Author contributions

SA carried out experimental work. AH supervise the study and finalize the MS. WM co-supervise the study. MH helped in phytohormones analysis. AI performed statistical analysis. MI helped in antioxidant analysis. HR and AT performed mass spectrometry. AA, AD, and HE provided facilities to perform phytohormones and antioxidant analysis. I-JL financially supported the study, provided facility, and experties for phytohormones analysis. All authors contributed to the article and approved the submitted version.

## Funding

This research was financially supported the Deanship of Scientific Research, King Saud University for funding through Vice Deanship of Scientific Research Chairs, Research Chair of Prince Sultan Bin Abdulaziz International Prize for Water and Korea Basic Science Institute (National research Facilites and Equipment center) grand funded by the Ministry of Education (2021R1A6C101A416).

## Conflict of interest

The authors declare that the research was conducted in the absence of any commercial or financial relationships that could be construed as a potential conflict of interest.

## Publisher's note

All claims expressed in this article are solely those of the authors and do not necessarily represent those of their affiliated organizations, or those of the publisher, the editors and the reviewers. Any product that may be evaluated in this article, or claim that may be made by its manufacturer, is not guaranteed or endorsed by the publisher.

## Supplementary material

The Supplementary Material for this article can be found online at: https://www.frontiersin.org/articles/10.3389/fpls.2022.922343/full#supplementary-material
