# Supplementary material for: Endophytic Fusarium oxysporum GW controlling weed and an effective biostimulant for wheat growth
Source: Front Plant Sci. 2022 Aug 5;13:922343. doi: 10.3389/fpls.2022.922343 (PMC9394004; doi:10.3389/fpls.2022.922343)
Supplement: Supplementary file 1 [file Data_Sheet_1.docx]

Table S1: Colonization frequencies of the isolated fungal strains in different parts of wheat plant and their impact of wheat and A. fatua (weed) growth.

| Strain | Leaf | Stem | Root | Wheat Promoting | Weed Inhibitor |
| --- | --- | --- | --- | --- | --- |
| GW (greyish white) | 70% | 60% | 90% | +++ | +++ |
| W1 (White 1) | - | - | 80% | +++ | ++ |
| W2 (White 2) | - | - | 100% | ++ | ++ |
| BK (Black) | 100% | - | - | N | ++ |
| YB (Yellowish Black) | 70% | 90% | 100% | + | GP |
| GR (Grey) | 80% | 100% | 80% | + | + |
| BR (Brown) | - | 80% | 70% | +++ | ++ |
| LG (Light Green) | 70% | - | - | ++ | N |

+++Highest, ++Middle, +Low, N= no visible effect on growth, GP=weed growth promotion

Table S2: Screening the HPLC fractions of *F. oxysporum* culture filtrate for inhibition of *Avena fatua* germination and growth

| Fractions | G% | MDG | VI | RL (cm) | SL (cm) | FW (g) | DW (g) |
| --- | --- | --- | --- | --- | --- | --- | --- |
| Control | 100 | 1.4 | 2150 | 9.5 ± 0,76 | 12 ± 1.1 | 0.38 ± 0.02 | 0.145 ± 0.012 |
| GW-A | 10 | 0.667 | 130 | 7 ± 0.81 | 6± 0.9 | 0.12 ± 0.01 | 0.0019 ± 0.0 |
| GW-B | 8 | 0.5 | 0 | 4.4 ± 0.27 | 4.5 ± 0.3 | 0.1 ± 0.01 | 0.0012 ± 0.0 |
| GW-C | 5 | 0.24 | 0 | 3.4 ± 0.31 | 5.3 ± 0.67 | 0.1 ± 0.008 | 0.001 ± 0.0 |

Figure S1: HPLC chromatogram showing three different fractions eluted from a reverse phase C18 column at 1.47 (A), 1.87 (B) and 8.40 (C) minutes through 70% methanol as mobile phase.

Figure S2: Determination of total phenols and flavonoids in the culture filtrate of Fusarium oxysporum GW grown in Czapek broth.


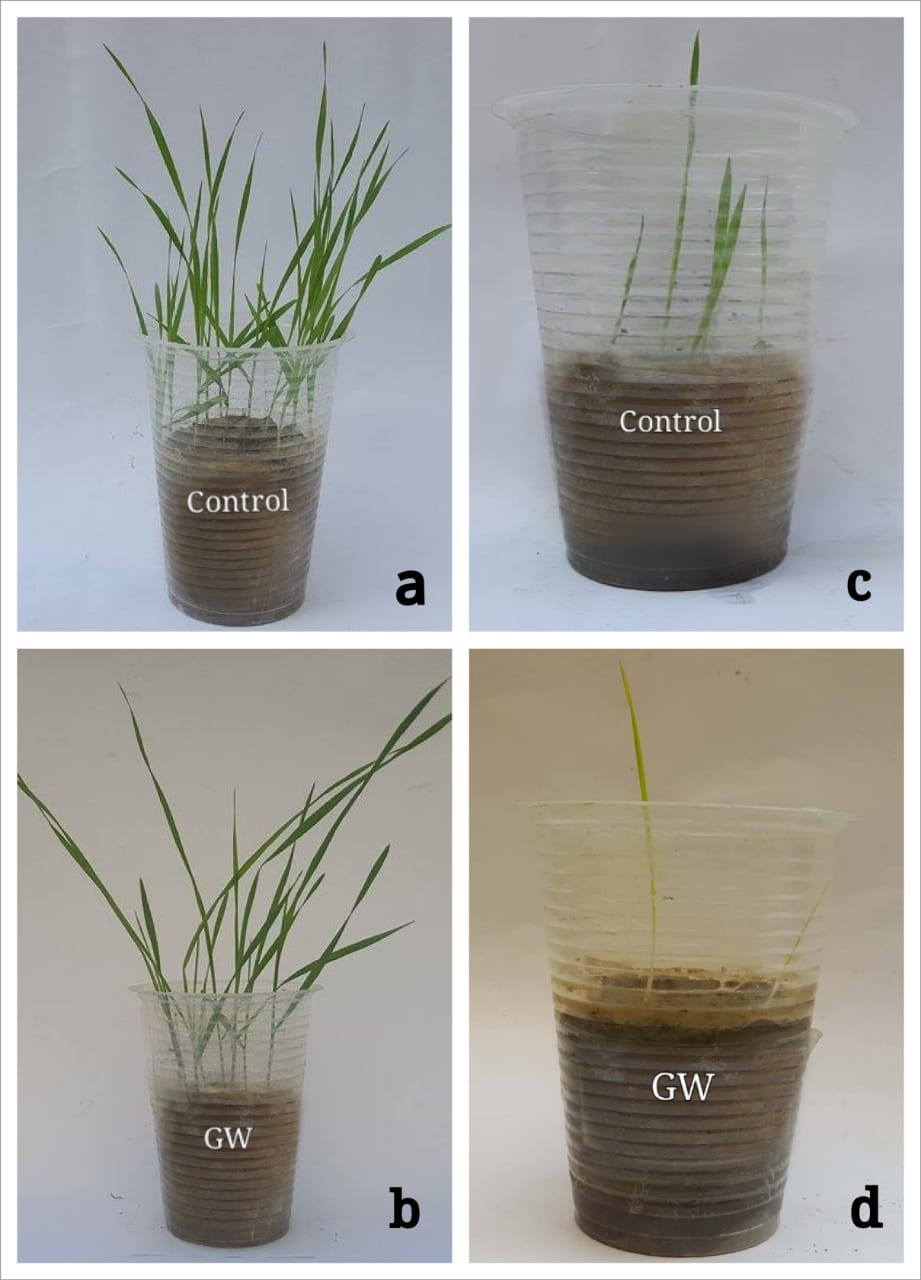


Figure S3: Effect of endophytic fungus GW on the growth of wheat (a and b) and *A. fatua* seedlings (c and d). Seedlings were grown in garden soil contained in plastic pots for 21 days.


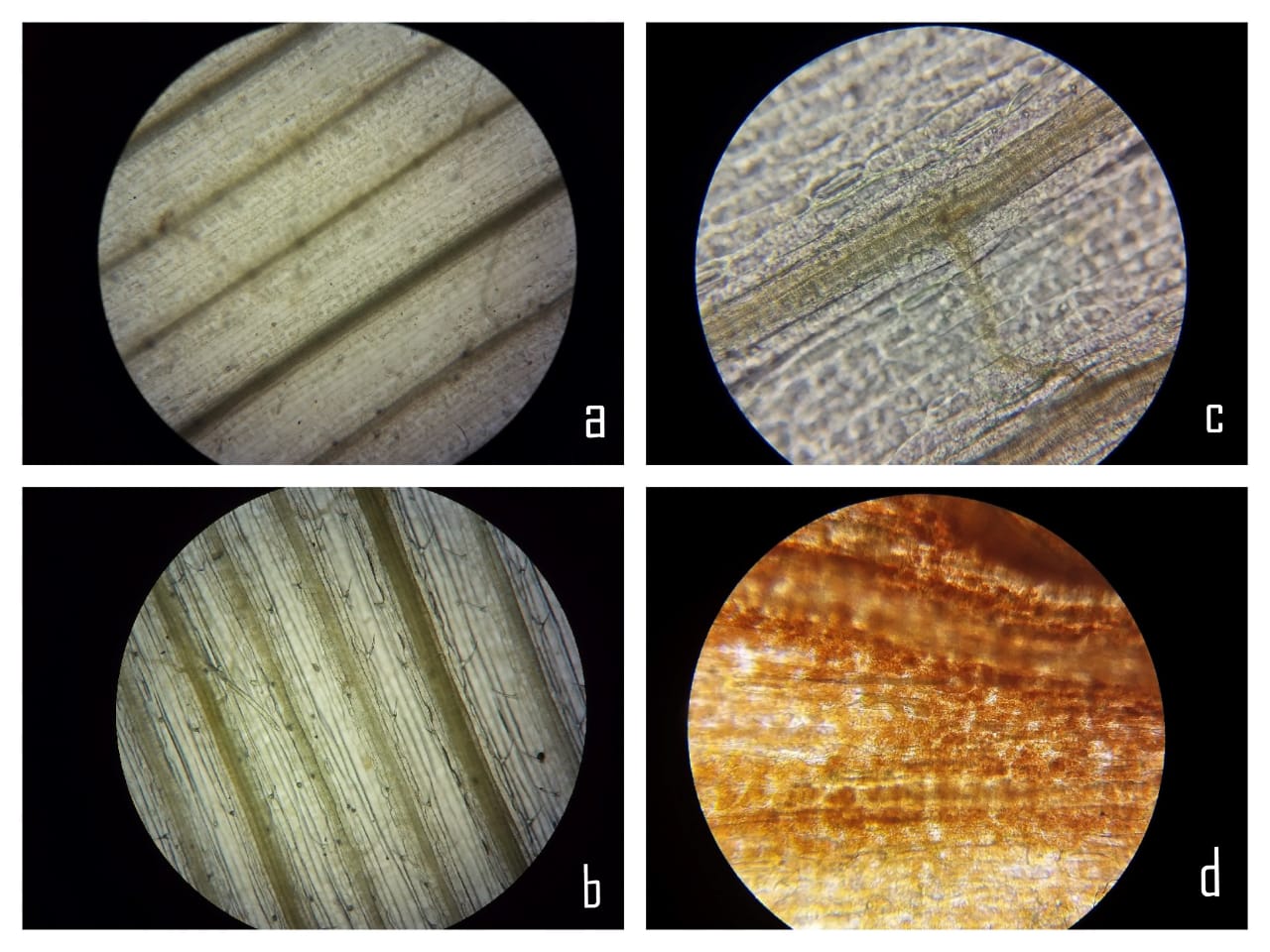


Figure S4: 3,3'-diaminobenzidine (DAB) assay for ROS accumulation in the leaves of wheat and *A. fatua* seedlings. Leaves obtained from (a) control wheat (b) inoculated wheat (c) *Avena fatua* control (d) Inoculated *avena fatua* were subjected to DAB staining and observed under light microscope at 100x magnification.


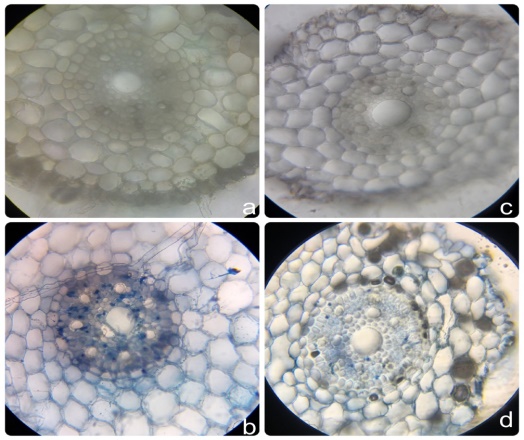


Figure S5. *Fusarium oxysporum* GW colonization observed in root section of (a) control wheat (b) GW inoculated wheat (c) control *Avena fatua* and (d) GW inoculated *A. fatua* seedlings. Lacto phenol cotton dye was used to stain the transverse sections of roots which were then observed under light microscope at 400X magnification.
